# Blood pressure time at target and its prognostic value for cardiovascular outcomes: a scoping review

**DOI:** 10.1038/s41440-024-01798-1

**Published:** 2024-07-16

**Authors:** Wansha Li, Sonali R. Gnanenthiran, Aletta E. Schutte, Isabella Tan

**Affiliations:** 1 School of Population Health, Faculty of Medicine and Health, University of New South Wales, Sydney, NSW Australia; 2 Department of Cardiology, Concord Hospital, Sydney, NSW Australia; 3 The George Institute for Global Health, University of New South Wales, Sydney, NSW Australia

**Keywords:** Blood pressure, Blood pressure variability, Hypertension, Time at target, Time in therapeutic range

## Abstract

The proportion of time that blood pressure (BP) readings are at treatment target levels, commonly referred to as time at target or time in therapeutic range (BP-TTR), is emerging as a useful measure for evaluating hypertension management effectiveness and assessing longitudinal BP control. However, method of determination for BP-TTR differs across studies. This review identifies variations in BP-TTR determination methodologies and its potential prognostic value for cardiovascular outcomes. Following PRISMA extension for scoping reviews guidelines, literature was systematically searched in Embase, PubMed, Scopus, Web of Science, and CINAHL. Relevant clinical trials, observational studies, cohort studies, cross-sectional studies, and systematic reviews published in English were screened. Of 369 articles identified, 17 articles were included. Studies differed in the BP targets used (e.g., BP < 140/90 mmHg or 130/80 mmHg; systolic BP within 110–130 mmHg or 120–140 mmHg), BP-TTR measurement duration (range 24 h to 15 years), and calculation method (linear interpolation method, *n* = 12 [71%]; proportion of BP readings at target, *n* = 5 [29%]). Regardless of method, studies consistently demonstrated that higher BP-TTR was associated with reduced risk of cardiovascular outcomes. Six of eight studies found the association was independent of mean achieved BP or last measured BP. Despite variation in methods of BP-TTR determination, these studies demonstrated the potential prognostic value of BP-TTR for cardiovascular outcomes beyond current BP control measures. We recommend standardization of BP-TTR methodology, with preference for linear interpolation method when BP measurements are few or less frequent, and proportion of BP readings method when large number of BP readings are available.

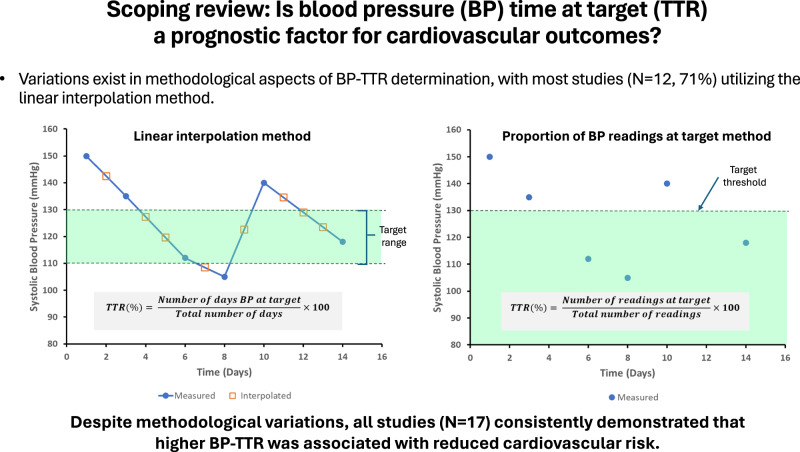

## Introduction

Raised blood pressure (BP) is strongly and directly related to cardiovascular outcomes and all-cause mortality, with high systolic BP responsible for over 10 million deaths per year, worldwide. According to World Health Organization data, an estimated 1.3 billion people worldwide have high BP, but only 14% of hypertensive patients achieve BP control [1]. Therefore, using an effective method for BP management is important to achieve treatment effectiveness and reduce adverse clinical events.

To date, BP management has mostly been based on BP measurements taken in a clinician’s office. However, such snapshot readings cannot provide the true picture of an individual’s BP control, particularly if visits to the clinician are far apart [2]. This can often lead to missed diagnosis of white coat hypertension or masked hypertension. Out-of-office BP monitoring such as ambulatory or home BP monitoring can provide additional information about an individual’s BP profile, but are still inadequate for capturing BP variations that reflect BP control when average BP readings are used for clinical decisions. This is because averaging of BP readings inevitably ‘evens out’ any fluctuations in BP that may convey important prognostic information [2]. Given surmounting evidence showing that increased fluctuations in BP over time increases cardiovascular risk [3–5], it is all the more important to have a measure that captures BP variability and control over time. Such a measure may also provide prognostic information above and beyond averaged BP readings over time.

The concept of “time in therapeutic range (abbreviated TTR)” originated from determining performance of oral anticoagulation therapy [6], but the term itself in relation to BP was first introduced by Doumas et al. [7] as a novel measure of hypertension management to capture BP variability. It represents the proportion of time an individual’s BP readings are within a specified range. Doumas et al.’s study showed that TTR, determined as the proportion of BP readings within target range (systolic BP within 120–140 mmHg) over 10 years, had an inverse and gradual association with all-cause mortality [7]. Not long after, the term BP “time at target (abbreviated TITRE)”, which is similar to TTR but calculated differently using a linear interpolation method [6] and target BP was a threshold rather than a narrow range, was introduced by Chung et al. [8]. Chung et al. similarly found an inverse association between TITRE and risk of incident cardiovascular diseases [8]. The two terms, TTR and TITRE, have since been used interchangeably by subsequent studies [9–11]. Despite the growing interest in the use of TTR/TITRE, there has yet to be a comprehensive review on TTR/TITRE. Furthermore, there is currently no standardized method for determining TTR/TITRE, making meta-analyses of results from TTR/TITRE studies difficult. This scoping review was thus conducted using the PRISMA extension for scoping reviews (PRISMA-ScR) [12] to: (1) identify and describe current TTR/TITRE measurement methodologies, including all factors that are used for the determination of TTR/TITRE; (2) evaluate the prognostic value of TTR/TITRE for cardiovascular outcomes. TTR and TITRE will be collectively referred to as BP-TTR for the current review.

## Methods

### Data sources and search strategy

Searches for relevant articles were performed in five relevant medical databases: Embase, PubMed, Scopus, Web of Science, and CINAHL. Articles published up until 27 September 2023 were considered without lower limit for publication year. The search terms encompassed MeSH terms as well as free-text terms relating to BP, TTR, and adverse cardiovascular and clinical outcomes. Details of specific search terms and search strategies used in each database can be found in the online only Supplement (Supplementary Table 1).

### Inclusion and exclusion criteria

Clinical trials, observational studies, cohort studies, cross-sectional studies, and systematic reviews related to BP-TTR and cardiovascular outcomes and all-cause mortality were included for screening. Only English language articles were included. Studies associated with anticoagulation dose management or not relevant to cardiovascular outcomes or all-cause mortality were excluded. Conference proceedings and abstract-only publications were also excluded.

### Study selection and data extraction

**Fig. 1:**
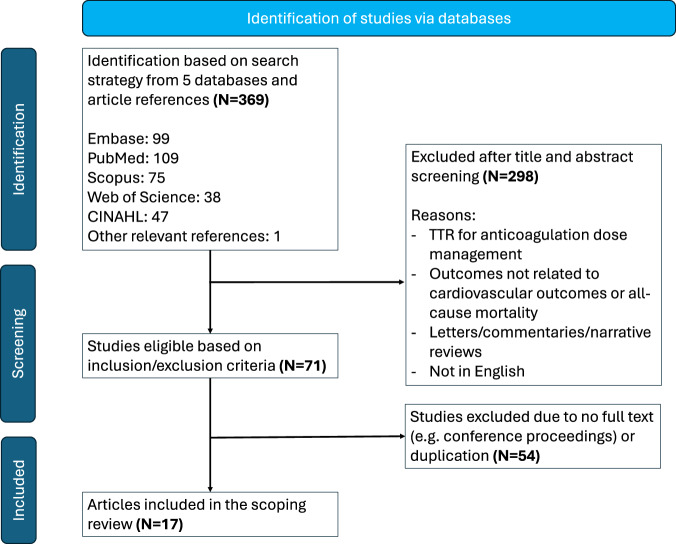
Flowchart representing the selection of sources of evidence

368 articles were identified by the literature search in the five databases listed above and 1 article was identified from relevant article references. After screening the titles and abstracts by two investigators (WL and IT), 298 articles were excluded after applying the inclusion–exclusion criteria. From the 71 articles that met the inclusion criteria, duplicates and articles without full texts were removed (*n* = 54), resulting in 17 studies included in the present scoping review (Fig. 1). Data items including document types, duration of study, sample size, biological sex, age, terminology for BP-TTR, BP target or target range, BP-TTR calculation method, mode of BP measurement, study objectives, cardiovascular outcomes, and study main findings were extracted from included studies. One investigator (WL) extracted the data information, and a second investigator (IT) checked these data for accuracy. The investigators discussed the indistinct points until agreement was reached.

## Results

### Study characteristics

Study characteristics of 8 clinical trials and 9 cohort studies, including study type, study cohort, study objectives and major findings are presented in Table 1. Studies consisted of post-hoc analysis of previously conducted clinical trials or retrospective analysis of data from existing health registries. Of note, three studies used the ACCORD BP trial cohort [13**–**15], two studies used the SPRINT cohort [13, 16], and two studies used the TOPCAT cohort [9, 17]. Studies involving the same trials had slightly different inclusion criteria and/or analyses, thus were all included in the present review. The predominant sex of included studies was male (average 64% of a total sample of 941,977) and the mean age was 60 ±13 years.

**Table 1:** Characteristics and main findings of included studies

Study	Study Type	Follow-up Duration	Cohort	*N*	Male (%)	Age (years)	Study Objectives	Main findings	Independent of BP?
Buckley et al. [13]	secondary analysis of SPRINT and ACCORD BP trials	SPRINT: Median 3.26 years ACCORD: Median 4.94 (IQR 4.14–5.69) years	SPRINT: Hypertensives without T2DM ACCORD: Hypertensives with T2DM (intensive BP target arm only)	10,047	63	67 ± 9	Association between SBP-TTR and kidney and cardiovascular events.	In fully adjusted models (including baseline SBP), **higher 3-month SBP-TTR** (70- < 100%) was **associated with a reduced risk of composite MACE** (HR 0.69 [0.52, 0.91]), CV death (0.5 [0.38, 0.98]), and **HF hospitalization** (0.46 [0.29, 0.73]) compared to SBP-TTR of 0%. Sensitivity analyses showed **higher 12-month SBP-TTR did not significantly associate with a lower risk** of adverse cardiovascular events. This was possibly due to shorter follow-up and fewer overall events. **Association between 3-month SBP-TTR and major kidney and cardiovascular events was attenuated (no longer associated)** when models were **adjusted for mean achieved SBP** as opposed to baseline SBP.	Yes (for composite MACE, HF hospitalization, and nonfatal stroke; independent of only baseline SBP but not mean achieved SBP).
Chen KY et al. [14]	post hoc analysis of the ACCORD BP trial	Median 4.94 (IQR 4.14–5.69) years	ACCORD: Hypertensives with T2DM	8907	61	63 ± 7	Association between SBP-TTR and composite MACE Predictive value of SBP-TTR with 5-year MACE risk	A **higher SBP-TTR** (62–100%) was **associated with a 46% reduction in MACE** (HR 0.53 [0.43, 0.66]) when compared to low SBP-TTR (0–23%) as well as for secondary outcomes (nonfatal stroke, nonfatal MI, HF, CV death). Per 1% SD increase in SBP-TTR was associated with a 11% decrease in MACE (HR 0.89 [0.87, 0.92]). **Changing SBP target range** to 120–130 mmHg showed **similar results** (HR 0.93 [0.89, 0.98]. **SBP-TTR** had a similar model performance to averaged achieved SBP and **remained a significant predictor of 5-year MACE even when adjusted for averaged SBP**.	Yes (baseline SBP, last SBP before event, or mean achieved SBP).
Chen KY et al. [9]	post hoc analysis of TOPCAT and BEST trials	TOPCAT: 3.3 years BEST: 2.0 years	TOPCAT: Hypertensive with HFpEF BEST: Hypertensive with HfrEF	4789	58	66 ± 11	Prognostic value of SBP-TTR in risk of MACE in hypertensive HF patients.	The **top quartile of SBP-TTR** (38–100%) was **significantly associated with a lower risk of primary outcome** (combined endpoint of CV death of HF hospitalization) (HR 0.71 [0.60, 0.82]) as well as all secondary outcomes (CV mortality, HF hospitalization, all-cause mortality and any hospitalization) in a dose dependent manner. Sensitivity analyses showed that **using a different SBP target range of 110–130** **mmHg did not change the associations**. DBP-TTR with a target range of 70–80 mmHg was also shown to be significantly associated with primary outcome, cardiovascular, and all-cause mortality.	Yes (baseline SBP)
Cheng et al. [15]	post hoc analysis of the ACCORD BP trial	4.9 (4.14–5.69) years	ACCORD: Hypertensives with T2DM	4651	52	63 ± 7	DBP-TTR and SBP-TTR as CV risk marker in hypertensive patients with T2DM.	**Per 1-SD increase in both DBP-TTR** (37%) and **SBP-TTR** (36%) was **significantly associated with lower risks of primary outcome** (composite nonfatal MI, nonfatal stroke, or CV death; HR for DBP-TTR 0.86 [0.77, 0.95]; HR for SBP-TTR 0.82 [0.75, 0.91]), independent of BP group, baseline DBP or SBP and mean DBP or SBP. Compared to TTR < 25%, both DBP-TTR ≥ 75% (HR 0.64[0.47, 0.87]) and SBP-TTR ≥ 75% (HR 0.65 [0.49, 0.86]) groups had decreased risk of primary outcome. DBP-TTR remained significantly associated with risk of nonfatal MI independent of baseline BP and mean BP, whereas SBP-TTR remained significantly associated with all secondary outcomes (all-cause mortality, CV death, CHF, nonfatal MI) except total stroke.	Yes (baseline BP and mean BP)
Chung et al. [8]	cohort study	13 years	newly identified hypertension without cardiovascular disease	169,082	44	52 ± 14	Association between BP-TTR and risk of CV events and mortality.	A **higher BP-TTR** (≥3 months, or 25%) was **associated with lower risk of primary outcomes** (composite of CV death, MI or stroke; HF; CV disease or CV death) in a graded, stepwise fashion compared to 0% BP-TTR (e.g., adjusted OR for composite outcome at BP-TTR 9 to <12 months: 0.26 [0.18, 0.36]). Sensitivity analyses showed a **weaker association of any CV disease with either snapshot BP control statuses** or **mean follow-up BP** compared to BP-TTR, and that the **number of follow-up BP measurements did not affect the association of BP-TTR with outcomes**.	Not reported (models were not adjusted for BP per se, but adjusted for stage 2 hypertension status and snapshot BP control status, defined as a single on-target BP measurement within first year of follow up)
Doumas et al. [7]	cohort study (VA)	12 years	hypertensive, intermediate hypertensive, and normotensive	689,051	94	62 ± 13	Association between SBP-TTR and all-cause mortality.	A **lower SBP-TTR** (<75%) was **associated with increased all-cause mortality rates** compared to SBP-TTR of 75–100% in a graded, stepwise fashion in both intermediate hypertensives (1–2 elevated BP readings) and established hypertensives (3 or more elevated BP readings). OR for all-cause mortality at SBP-TTR 0–25%: 2.97 [2.80, 3.16] in established hypertensives; OR 2.67 [2.49, 2.86] in intermediate hypertensives. Cox regression for the whole cohort showed highest HR for mortality with SBP-TTR 0–25% (2.18 [2.06–2.32]), with little difference between SBP-TTR between 50–75% and 75–100%.	Not reported (models were not adjusted for BP, but analyses were performed in groups with different BP categories).
Fatani et al. [16]	post hoc analysis of the SPRINT trial	3.3 years	adults with hypertension without diabetes or prior stroke	9361	62	68 ± 10	Association between SBP-TTR and composite MACE in hypertensive patients.	**Per 1-SD (40%) increase in SBP-TTR was significantly associated with a decreased risk of first major adverse CV events** (composite CV death, MI, non-MI ACS, stroke, or acute decompensated HF; HR 0.85 [0.74, 0.96]) in fully adjusted models. Sensitivity analysis showed mean SBP was not associated with CV outcomes when stratified by SBP-TTR groups. **Extending period of SBP-TTR from 3 months to 6 months resulted in loss of significant association with MACE, but associations with CV and all-cause mortality remained**.	Yes (baseline SBP, mean SBP).
Fu et al. [22]	post-hoc analysis of STICH trial	8.9 years	patients with ischemic cardiomyopathy (61% hypertensive)	1194	87	60 ± 14	Association between SBP-TTR and composite MACE in patients with ischemic cardiomyopathy.	**Per 1-SD (29%) decrease in SBP-TTR significantly elevated the risk** of CV mortality (HR 1.15 [1.02–1.29]), all-cause mortality (1.15 [1.04–1.26]) and the combined risk of all-cause mortality plus CV rehospitalization (1.14 [1.06–1.23]) in fully adjusted model. SBP-TTR of 0–33% had higher risk of all-cause mortality compared to SBP-TTR of 78–100% (HR 1.53 [1.14, 2.04]). Sensitivity analyses showed **changing SBP target range to 110–140** **mmHg or adding mean SBP produced similar results** for associations between SBP-TTR and outcomes.	Yes (baseline SBP, mean SBP)
Huang et al. [17]	a secondary analysis of the TOPCAT trial	3 years	HFpEF (91% hypertensive)	3194	49	68 ± 10	Association between SBP-TTR and adverse clinical events in patients with HFpEF.	Per 1-SD (38%) **increase in SBP-TTR was associated with a decreased risk of primary CV outcome** (composite of CV death, aborted cardiac arrest, or HF hospitalization; HR 0.80 [0.72, 0.88]), all-cause mortality (0.79 [0.71, 0.88]), CV death (0.77 [0.67, 0.88]) and HF hospitalization (0.83 [0.73, 0.94]). Sensitivity analysis showed **extending BP-TTR time period from 4 months to 12 months did not change association** between BP-TTR and primary outcome (0.81 [0.71–0.92]).	Yes (mean SBP)
Kakaletsis et al. [23]	cohort study (PREVISE)	3 months	acute ischemic stroke patients (84% hypertensive)	228	46	80 ± 7	Association of short-term (24-h) BP-TTR with stroke outcomes.	Every 1% **increase in DBP-TTR** (OR 0.94 [0.90, 0.98]) **or MAP-TTR** (0.96 [0.93, 0.99]), but not SBP-TTR, was **significantly associated with a decreased risk of stroke-related disability/death**.	Yes (admission DBP and MAP, but not SBP nor mean DBP)
Kario et al. [19]	a secondary analysis of J-HOP study	6.3 years	patients with AF (79% hypertensive)	4070	54	65 ± 11	Association of SBP-TTR with CV events.	Compared to SBP-TTR of 100%, **SBP-TTR of** <**15.3% had a higher risk of total CV events** (HR 1.74 [1.15, 2.61]) **and stroke** (2.11 [1.06, 4.21])), but not CAD and HF. **A 10% decrease in home SBP-TTR was associated with a 4% increase in the risk of total CV events** (HR 1.04 [1.00, 1.08]) and a 9% increase in the risk of stroke (1.09 [1.03, 1.16]). Home SBP-TTR of ≥67% had reduced cumulative incidence of stroke.	Yes (office SBP)
Kim et al. [11]	cohort study	Median 2.7 years (IQR 1.1–4.9 years)	hypertensive patients with AF	9505	91	65 ± 12	Association of BP-TTR with thromboembolic events and ischemic stroke.	**SBP-TTR** < **94%** (HR 1.24 [1.00–1.52] at SBP-TTR between 69 and 94%; 1.55 [1.27, 1.89] with SBP-TTR between 39 and 69%) and **DBP-TTR** < **95%** (1.22 [1.01–1.48] with DBP-TTR between 77 and 95%) were **associated with an increased incidence rate of ischemic stroke** or systemic embolism in a stepwise manner. The best cut-off value of SBP-TTR to predict the risk of ischemic stroke and systemic embolism was 77%. **Those with SBP-TTR** ≥ **94% was associated with the lowest risk, comparable with normotensives**.	Not reported.
Kodani et al. [24]	a post hoc analysis of J-RHYTHM registry	2 years	Patients with nonvalvular AF (60% hypertensive)	7226	71	70 ± 10	Association of SBP-TTR with adverse outcomes, including CV death.	Each 1% **increase in SBP-TTR** (based on target range of 110–130 mmHg and included as a continuous variable) **was significantly associated with a decreased incidence of CV death** (HR 0.983 [0.974, 0.993]) and **all-cause death** (0.993 [0.987, 0.998]), in adjusted models. Lower SBP-TTR (based on target range of 110–130 mmHg) < 50% was associated with higher risk of CV and all-cause death compared with SBP-TTR ≥ 75%. **SBP-TTR based on a target range of 120–140** **mmHg was not associated with any adverse events in adjusted models** (regardless of whether SBP-TTR was treated as a continuous or categorical variable).	Yes (BP at time closest to event or end of follow-up).
Lin et al. [25]	cohort study	15 years	elderly participants with hypertension aged over 75 years	943	98	78 ± 5	Predictive value of SBP-TTR with future CV risks.	**For each 1-SD increase in SBP-TTR the risk of primary outcome** (composite stroke, MI, angina pectoris, or CV death) **decreased** by 25% (HR 0.75 [0.67, 0.83]). **Greater long-term SBP-TTR** ≥ 25% (with target range of 120–140 mmHg) was **associated with a decreased risk of primary outcome,** **regardless of the number of SBP readings** (5–8 compared to 6–16). HR for SBP-TTR of 75–100%, compared to SBP-TTR < 25%, was 0.42 [0.29, 0.62]. **Results were similar when a target range of** <**140** **mmHg was used**.	Yes (baseline SBP, last SBP and mean SBP)
Mahfoud et al. [20]	a post-hoc analysis of the Global SYMPLICITY Registry)	845 ± 383 days	Patients with uncontrolled hypertension who received renal denervation	3077	58	61 ± 12	Predictive value of SBP-TTR with future CV risks after renal denervation.	**A 10% increase in 6-month SBP-TTR was associated with significant risk reductions of 15% MACE** (combined CV death, MI, or stroke; HR 0.85 [0.79, 0.91]), 11% CV-death (0.89 [0.81, 0.97]), 15% MI (0.85 [0.75, 0.98]), and 23% stroke (0.77 [0.68, 0.88]).	Yes (baseline SBP, which was not predictive in the model).
^a^Mancia et al. [18]	a post-hoc analysis of VALUE trial	4–6 years	high CV risk patients with hypertension	15,244	58	67 ± 8	Association between SBP-TTR with incidence and risk of CV events.	**BP-TTR increase** (target range BP < 140/90 mmHg) was **accompanied by a significant decrease in the risk of CV morbidity and mortality**, MI, HF, stroke and all-cause mortality, with **greatest risk reduction when BP-TTR increased from** <**25% to 24–49% with modest to no decrease in risk as BP-TTR increases** (HR for composite total MI, sudden cardiac death, death from revascularization procedures, HF hospitalization, and emergency for MI prevention: 0.56 [0.49, 0.65]). **BP-TTR using a target range BP** < **130/80** **mmHg) showed no significant risk improvement compared to target range of BP** < **140/90** **mmHg**.	Yes (baseline SBP and DBP).
Sideris et al. [21]	cohort study	6 ± 3.3 years	hypertension without CV disease	1408	48	60 ± 11	Association of SBP-TTR with future CV events.	**1% increase of SBP-TTR** was **associated with 2% lower outcome** (composite major fatal or nonfatal CV events) (HR 0.98 [0.96, 0.99]). Lower SBP-TTR (≤25%) was associated with higher risk of outcome compared to SBP-TTR > 67% (HR 2.77 [1.40, 5.49]).out	Yes (baseline SBP and DBP)

*AF* atrial fibrillation, *BP* blood pressure, *CAD* coronary artery disease, *CV* cardiovascular, *DBP* diastolic blood pressure, *HF* heart failure, *HFpEF* heart failure with preserved ejection fraction, *HFrEF* heart failure with reduced ejection fraction, *HR* hazard ratio, *MACE* major adverse cardiovascular events, *MI* myocardial infarction, *OR* odds ratio, *SBP* systolic blood pressure, *T2DM* Type 2 diabetes mellitus, *TTR* time in target range

^a^Study identified from article references

### Terminology for BP-TTR

Aside from the study by Chung et al. [8], which used the term “time at target”, and Mancia et al. [18], which used the term “percentage of on-treatment visits”, all other included studies used the term “time in therapeutic range” (*n* = 5) [7, 11, 19–21] or “time in target range” (*n* = 10) [9, 13–17, 22–25] (Table 2).

**Table 2:** Terminology and methodological aspects for BP-TTR determination

Study	Term and abbreviation used	BP Measurement	Method	Time range	Frequency of BP measurements over BP-TTR time range	No. of BP readings	Target BP range
Buckley et al. [13]	Time in target range (TTR)	Office BP	linear interpolation	3 months	Monthly	2–4^a^	SBP: 110–130 mmHg (intensive); 120–140 mmHg (standard)
Chen KY et al. [14]	Time in target range (TIR)	Office BP	linear interpolation	4.94 (4.14–5.69) years (across the whole follow-up period)	Intensive arm: monthly for 4 months, then once every 2 months thereafter; Standard arm: 1 month, 4 month, and once every 4 months thereafter	15 (15–20) [Median (IQR)] (at least 3)	SBP: 110–130 mmHg
Chen KY et al. [9]	Time in target range (TTR)	Office BP	linear interpolation	TOPCAT: mean 3.3 years (across whole study period) BEST: mean 2.0 years (across study period)	TOPCAT: Month 0, 1, 2, 4, 8, 12, then every 6 months thereafter Best:0, 3, 6 months, and every 6 months thereafter	TOPCAT: 11 (range 2–16) BEST: 13 (range 2–28)	SBP: 120–130 mmHg
Cheng et al. [15]	Time in target range (TTR)	Office BP	linear interpolation	4 months	Monthly	Standard: 2–3^a^ Intensive: 2–5^a^	SBP: 110–130 mmHg (intensive); 120–140 mmHg (standard) DBP: 70–80 mmHg
Chung et al. [8]	Time at target (TITRE)	Office BP	linear interpolation	**annual** TTR was averaged over the whole period, which was median 4.9 years (IQR 2.6–7.3 years)	Not specified (based on primary care data)	Average 1.6 of BP measures per year	BP: <150/90 mmHg for those over 60 years without diabetes or CKD; <140/90 mmHg for all others
Doumas et al. [7]	Time in therapeutic range (TTR)	Office BP	proportion of BP readings	10 years	Not specified (based on electronic medical records)	Hypertensives: 31.9 ± 25.9 Intermediate hypertensives: 8.1 ± 6.1 Normotensives: 6.6 ± 4.8	SBP: 120–140 mmHg
Fatani et al. [16]	Time in target range (TTR)	Office BP	linear interpolation	3 months	Monthly	2–4^a^	SBP:110–130 mmHg (intensive); 120–140 mmHg (standard)
Fu et al. [22]	Time in target range (TTR)	Office BP	linear interpolation	8.9 years	4-month intervals during the first year, and at least every 6 months throughout the trial period.	Average 10 readings (minimum 5, maximum 28)	SBP: 110–130 mmHg
Huang et al. [17]	Time in target range (TTR)	Office BP	linear interpolation	4 months	at baseline, 4 weeks, 8 weeks, 4 months	3–4^a^	SBP: 110–130 mmHg
Kakaletsis et al. [23]	Time in target range (TTR)	Ambulatory BP	proportion of BP readings	24 h	every 20 min over 24 h	Approximately 66 per person	SBP: 90–140 mmHg DBP: 60–90 mmHg MAP: 70–105 mmHg
Kario et al. [19]	Time in therapeutic range (TTR)	Home BP	proportion of BP readings	13 days	Daily (average of morning and evening readings used)	5–13^a^	SBP: 100–135 mmHg
Kim et al. [11]	Time in therapeutic range (TTR)	Office BP	linear interpolation	2.7 years	Not specified (based on electronic medical records)	Median 14 (IQR 6–25)	SBP: <130 mmHg DBP < 80 mmHg
Kodani et al. [24]	Time in target range (TTR)	Office BP	linear interpolation	2 years	Not specified (BP was measured at least 4 times during the 2-year follow-up period or until occurrence of an event)	15 ± 5	SBP: 110–130 mmHg or 120–140 mmHg
Lin et al. [25]	Time in target range (TTR)	Office BP	linear interpolation	15 years	Yearly	3–15^a^	SBP: 120–140 mmHg
Mahfoud et al. [20]	Time in therapeutic range (TTR)	Office BP and Ambulatory BP	linear interpolation	6 months	Every 3 months	3^a^	Office SBP: ≤140 mmHg; Ambulatory 24-h SBP: ≤130 mmHg
Mancia et al. [18]	Percentage of on-treatment visits^b^	Office BP	proportion of BP readings	4–6 years	Monthly during the initial 6 months of treatment, 6 monthly thereafter.	1–17^a^	BP: <140/90 or <130/80 mmHg
Sideris et al. [21]	Time in therapeutic range (TTR)	Office BP	proportion of BP readings	6 ± 3.3 years	BP was measured at baseline and at least three visits in follow-up period, minimum one per year	4.9 ± 2.6	SBP: 120–140 mmHg

*BEST* Beta-Blocker Evaluation of Survivor Trial, *BP* blood pressure, *BP-TTR* blood pressure time at target/time in therapeutic range, *DBP* diastolic blood pressure, *IQR* interquartile range, *SBP* systolic blood pressure, *TOPCAT* Treatment of Preserved Cardiac Function with Heart Failure With an Aldosterone Antagonist Trial

^a^Number of BP readings was not specified, maximum number in range determined based on reported TTR calculation duration and frequency of BP measurements

^b^Although this study used the term “percentage of on-treatment visits”, its determination is essentially the same as proportion of BP readings at target, hence included as such

### Methodology for BP-TTR determination

Methodology for BP-TTR determination, including mode and frequency of BP measurements, calculation methods, and the duration over which BP-TTR was determined, are presented in Table 2, with an overall summary of variations presented in Supplementary Table 2.

#### Mode of BP measurements

Office BP was the dominant BP measurement (*n* = 15), taken by either automated blood pressure devices [8, 9, 11, 13–17, 19–22, 24] or manually with a mercury sphygmomanometer [18, 25]. The J-HOP study recorded home BP obtained by a validated cuff oscillometer home BP monitoring device [19]. Ambulatory 24-h BP monitoring was performed in two studies [20, 23].

#### Frequency and total number of BP measurements

Frequency of BP measurements used for BP-TTR determination was dependent on BP measurement modality as well as the clinical trial protocol or standard clinical care (for health registries). Clinical trials or cohort studies with office BP as the BP measurement modality had BP taken at least monthly, with the frequency ranging from monthly to once per year (Table 2). Studies using primary care data [7, 8, 11, 25] had much less frequent BP measurements. The duration over which BP-TTR was determined also differed among the studies, ranging from very short term [19, 23] (24 h for ambulatory BP and 13 days for home BP), short term (3–6 months) [13, 15–17, 20], and longer term (1–15 years) [7–9, 11, 14, 18, 21, 22, 24, 25] (Table 2). Total number of BP measurements used for BP-TTR determination was dependent on both frequency of BP measurements and the duration over which BP-TTR was determined and ranged from a minimum of 2 measurements to as many as 32 readings. The study that used ambulatory BP over 24 h for determining BP-TTR [23] used on average 66 readings per person to determine BP-TTR, as BP was measured every 20 min. The study that used home BP over 13 days [19] had participants measuring BP twice a day, and 5–13 readings were used for determining BP-TTR.

#### BP target

Six studies [13, 15, 16, 24] adopted two systolic BP target ranges for determining BP-TTR, four of which used a systolic BP target range of 120–140 mmHg for standard group and 110–130 mmHg for intensive group according to the SPRINT protocol. Four studies [8, 11, 18, 20] used a target BP level as opposed to a BP range, with some using different cut-offs for different groups (e.g., Chung et al. [8] used BP < 150/90 mmHg for adults aged above 60 years without diabetes and chronic kidney disease but BP < 140/90 mmHg for all others). The target BP levels or ranges chosen by different studies were also dependent on the mode of BP measurements in accordance with hypertension management guidelines. It is noted that most studies only evaluated systolic BP-TTR, with three studies also including a diastolic BP-TTR [11, 15, 23]. Three studies [8, 18, 20] determined BP-TTR with both SBP and DBP considered together [8, 18].

#### BP-TTR calculation methods

**Fig. 2:**
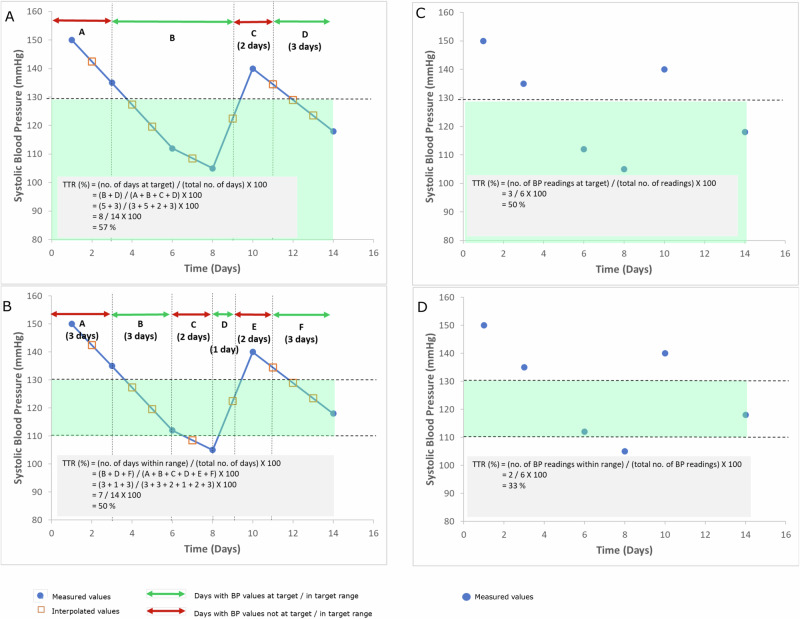
Schematic representation of the two methods for determining BP-TTR. In this schematic representation, the time duration over which BP-TTR is determined is 14 days and BP target level is systolic BP < 130 mmHg, or systolic BP within 110–130 mmHg. All four figures show the same data. The left (**A**, **B**), represents the linear interpolation method, which assumes a linear change between two consecutive BP measurements (i.e., BP changes the same amount with each unit change in time; closed circles) and BP between the consecutive measurements are interpolated (open squares). BP-TTR is determined as the proportion of time BP was (**A**) below the target level, or (**B**) within the target range, i.e., the number of days BP was in range divided by the total number of days over which BP-TTR is determined. The right (**C**, **D**), represents the proportion of BP readings method. BP-TTR is determined as the proportion of BP readings (**C**) below the target level, or (**D**) within the target range (bottom graph), i.e., the number of BP readings in range divided by the total number of BP readings within the duration over which BP-TTR is determined

Studies employed two different methods for determining BP-TTR (see Table 2). Most studies adopted the linear interpolation method (*n* = 12, 71%), which assumes a linear change between two consecutive BP measurements and BP values in between the consecutive measurements are interpolated. This method assumes BP changes the same amount with each unit change in time (usually in terms of days), and BP-TTR is then determined as the proportion of time during which BP is below the set target level or within the set target range (Fig. 2, left panel). Other studies (n = 5, 29%) adopted the proportion of BP readings as the BP-TTR calculation method. This method determines BP-TTR simply as the proportion of BP measurements that is below the set target level or within the set target range (Fig. 2, right panel). Of note, the study by Mancia et al. [18] used the term “proportion of on-treatment visits”, but its determination is essentially the same as proportion of BP readings. Contrary to the original usage of the terminology by Doumas et al. [7], of the 15 studies that used the term “time in therapeutic range” or “time in target range”, two studies [11, 20] used a BP target level as opposed to a narrow range, and only three studies [19, 21, 23] used the proportion of BP readings method. The method used in Chung et al.’s study [8] also slightly differed from others in that an annual BP-TTR was calculated then averaged over the whole follow-up period, rather than the whole follow-up period considered as a whole.

### Association of BP-TTR and cardiovascular outcomes

The major findings from each of the included studies are summarized in Table 1. Where reported, the hazard ratios or odd ratios for associations between BP-TTR and cardiovascular outcomes are also presented. Due to the heterogeneity of the studies, a meta-analysis was not performed. The key cardiovascular outcomes were composite major adverse cardiovascular events (MACE), nonfatal or fatal myocardial infarction (MI), nonfatal or fatal stroke, and cardiovascular death or all-cause mortality (for full details of the cardiovascular outcomes in each study, refer to Supplementary Table 3 in the online only Supplement).

All studies consistently demonstrated that a higher BP-TTR was associated with lower risk of cardiovascular outcomes, with reduction of risk ranging from 5% [16, 17] to 20% [21] per 10% increase in office BP-TTR (Table 1). In all studies where baseline BP was included in study models (n = 11), the association between BP-TTR and cardiovascular outcomes was independent of baseline BP. However, results were variable when models were adjusted for mean achieved BP or last measured BP (Table 1, Supplementary Table 4). Chen et al. [9] and Lin et al. [25] found that greater systolic BP-TTR was associated with a decreased risk of nonfatal MI, nonfatal stroke, and cardiovascular death in elderly individuals, independent of mean BP. The study by Kodani et al. [24] also found that systolic BP-TTR remained associated with cardiovascular and all-cause mortality even when adjusted for BP closest to event or at end of follow-up period. Contrarily, Buckley et al. [13] found that the association between systolic BP-TTR and MACE was attenuated and no longer significant when models were adjusted for mean achieved SBP.

The association of BP-TTR and adverse cardiovascular outcomes appear to also be dependent on the target BP range selected. For example, some studies showed that the association between systolic BP-TTR with cardiovascular outcomes was similar between different target ranges (e.g., target BP range of 110–130 mmHg compared with 120–140 mmHg [18], or 120–140 mmHg compared with <140 mmHg [25]), but the study by Kodani et al. showed that only BP-TTR determined from a target BP range of 110–130 mmHg (and not 120–140 mmHg) had significant association with cardiovascular death [24].

Only one study [14] conducted prediction analysis and found that systolic BP-TTR was predictive of 5-year risk of MACE even in the presence of mean achieved systolic BP.

### Threshold of BP-TTR for cardiovascular risk reduction

Whilst all studies showed a linear relationship (TTR is analyzed as a continuous variable) or stepwise progression (when BP-TTR is analyzed as categorical groups) relationship between BP-TTR and risk of cardiovascular outcomes, studies differed in the threshold value (or range) beyond which there either is little to no more improvement of risk reduction, or improvement is not observed until BP-TTR has reached beyond that point (Supplementary Table 5). For studies using office BP (see Table 2), a number of studies found there little to not much difference in risk reduction beyond BP-TTR of 19% [9], 50-55% [7, 8, 22, 25], to 77% [11]. However, other studies showed improvement in risk reduction was not observed until BP-TTR reached above 70% [13] or 75% [15, 17] (Supplementary Table 4). Still others showed continued improvement of risk reduction from a BP-TTR of 23% up to a BP-TTR of 100% [14]. One study found the cut-off value of home systolic BP-TTR for reducing the risk of stroke (fatal and nonfatal) was 67% [19].

## Discussion

The main aims of this scoping review were to identify and describe different BP-TTR calculation methods and evaluate the prognostic value of BP-TTR for cardiovascular outcomes. The results showed that BP-TTR determination methodology was not consistent across studies in relation to the calculation method, BP targets, duration over which BP-TTR was determined, and number of BP readings. Despite the heterogeneity, higher BP-TTR was consistently associated with reduced cardiovascular outcomes in all studies. Six of eight studies that investigated the additive value of BP-TTR in the presence of mean or last achieved BP found independent association of BP-TTR with cardiovascular outcomes.

### Methodology variations for determining BP-TTR

There currently exists two general methods for BP-TTR calculation. The primary method used for BP-TTR determination was the linear interpolation method, which is based on the Rosendaal linear interpolation method [6] used for determining internal normalized ratio (INR)-specific incidence rates of untoward events to determine the optimal achieved intensity of anticoagulation. The alternative method was to determine BP-TTR as the proportion of BP readings in target range. Whilst the proportion of BP readings can be calculated more easily, the resultant BP-TTR doesn’t accurately reflect the time component of BP control. By definition, the proportion of BP readings in target range would result in a different BP-TTR as compared to the BP-TTR calculated using linear interpolation method for the same set of data, unless BP was measured daily or very close together in time. When the BP measurement interval is longer, the deviation between the two methods becomes larger. On the other hand, the linear interpolation method is more difficult and requires specialized software for calculation. It is also more susceptible to bias in the presence of extreme out-of-range values [26]. Studies comparing TTR determination methods for INRs have shown that the Rosendaal linear interpolation method has a tendency of resulting in lower TTR values compared to the proportion of INR at target method [27], with a high degree of variability between the two methods [27]. It is thus difficult to determine whether one method is more superior than the other, and the method to use may be dependent on mode and frequency of BP measurements. For example, it may be more appropriate to use linear interpolation method for office BP readings over months or years, as the interval between readings is long. However, for home BP, ambulatory BP, or cuffless BP that provide daily or more frequent BP readings, proportion of BP readings at target would be a simpler and more practical method to use.

Other variations in BP-TTR methodology such as duration over which BP-TTR is determined, frequency and number of BP measurements, as well as target BP range, are mainly dependent on the mode of BP measurement employed.

### Prognostic value of BP-TTR for cardiovascular outcomes and all-cause mortality

All included studies, regardless of methodology used for determining BP-TTR, consistently showed that a higher BP-TTR resulted in a lower risk of cardiovascular outcomes and/or all-cause mortality. However, sensitivity analyses from these studies also demonstrated that each methodology aspect of BP-TTR determination, including frequency and number of BP measurements, and duration of interest can affect the association between BP-TTR and cardiovascular outcomes. For example, Buckley et al. demonstrated that whilst a higher 3-month TTR was significantly associated with a lower risk of adverse cardiovascular event, the association was diminished when 12-month TTR was used [13]. However, this may have been specific to this study as there were less number of follow-ups and fewer overall events beyond the 3-month period [13]. Fantani et al. [16] also showed that extending the duration from 3 months to 6 months resulted in loss of significant association between systolic BP-TTR and MACE, but associations with cardiovascular mortality remained. Contrarily, Huang et al. [17] showed that extending the time period from 4 months to 12 months did not change the significant association between systolic BP-TTR and composite cardiovascular outcome. Chung et al. [8] similarly showed that the average number of BP readings did not affect the association between BP-TTR and outcomes.

The target BP range used in the determination of BP-TTR may also influence the prognostic value of BP-TTR. However, of studies that performed sensitivity analyses to determine whether different target BP ranges would affect the association between BP-TTR and cardiovascular outcomes, only one study found that using a target BP of 120–140 mmHg resulted in the disappearance of the association as opposed to a target BP of 110–130 mmHg [24] (Table 1). Of note, majority of the included studies used systolic BP only for BP-TTR, with some studies also using diastolic BP. Only Chung et al. [8] and Mancia et al. [18] considered systolic and diastolic BP together. Given the linear associations between systolic BP and cardiovascular risk but a J-curve relationship between diastolic BP and cardiovascular risk, there may be merit in separating systolic and diastolic BP-TTR. Furthermore, it may be helpful to determine whether using a target range for diastolic BP would be more suitable than using a singular target threshold when determining the prognostic value of diastolic BP-TTR with cardiovascular outcomes.

Given the wide range of methodological aspects in BP-TTR determination, it was not possible to converge on a specific threshold that optimizes cardiovascular risk reduction, with some studies showing risk reduction is only achieved beyond BP-TTR > 70% [13], whilst others showed risk reduction begins even at low BP-TTR (e.g., 19% [9]). Nevertheless, the consistent message of all studies is that a higher BP-TTR lowers risk of adverse cardiovascular outcomes. Given the availability of study cohorts with large number of participants, a systematic comparison of the different methods within the sample cohort may provide further insight on whether standardizing a method for BP-TTR determination is feasible and practical. It should be noted that all included studies are retrospective analyses, and prospective studies using BP-TTR as an outcome measure or as a predictor of cardiovascular outcomes are yet to be published at the time of this review. However, there is at present an ongoing prospective study that uses BP-TTR < 90%, where BP is measured using a wearable cuffless BP device, BP target is set at <135/85 mmHg, and BP-TTR is determined over 7 days using the proportion of BP readings method, as an indication for BP control and medication titration [28].

### Limitations

This scoping review aimed to identify the different methodologies used for determining BP-TTR and to evaluate, specifically, its prognostic value for adverse cardiovascular outcomes and all-cause mortality. The search strategies and inclusion/exclusion criteria for cardiovascular outcomes and all-cause mortality were therefore strictly applied at the initial extraction stage, but this may have reduced sensitivity of the paper extraction in terms of other utility aspects of BP-TTR, such as its use as an outcome measure.

## Conclusions

The present review demonstrated that variations exist in all factors used for the determination of BP-TTR, including calculation method, BP target range, number of BP measurements, and duration. This can make it difficult to compare BP-TTR across studies, particularly for meta-analyses. Despite this heterogeneity, studies included in the present review showed that both lower short-term and lower long-term BP-TTR were associated with adverse cardiovascular outcomes and that higher BP-TTR was associated with reduced cardiovascular risk. Six of eight studies also showed that this association was independent of last measured BP or mean BP, both measures used for assessing BP control. Although it may be impractical to standardize all aspects of BP-TTR determination, for example, duration over which BP-TTR is determined, it may be helpful to have a consensus on the calculation method (whether linear interpolation or proportion of at-target readings is preferable) and BP target range (for example, to align with treatment target in clinical practice). From a practicality standpoint, we recommend that linear interpolation method be used when BP readings are few and/or taken far apart in time (e.g., with office BP), but to use proportion of BP readings at target when a large number of BP measurements are available over time (e.g., with home BP, ambulatory BP, or cuffless BP). We encourage investigators to fully disclose all methodological aspects when reporting BP-TTR in order to enable informed comparisons across trials. When consensus of BP-TTR determination can be reached, further research can then determine practical thresholds for BP-TTR in clinical practice for optimal BP control and cardiovascular risk management. As included studies were all retrospective analyses, prospective studies will further inform the prognostic value for BP-TTR.

## Supplementary information

Supplementary Material
