# Supplementary material for: Blood pressure time at target and its prognostic value for cardiovascular outcomes: a scoping review
Source: Hypertens Res. 2024 Jul 16;47(9):2337–50. doi: 10.1038/s41440-024-01798-1 (PMC11374670; doi:10.1038/s41440-024-01798-1)
Supplement: Supplementary file 1 — Supplementary Material [file 41440_2024_1798_MOESM1_ESM.pdf]

Blood pressure time-at-target and its prognostic value for cardiovascular outcomes: a scoping review

Li W, Gnanenthiran SR, Schutte AE, Tan I.

Supplementary Material

Supplementary Table 1. Search strategies.

Database	Search strategy – e.g. keywords, limiters	Search date	No. of results	Comments
Embase	(hypertension/ or systolic hypertension/ or arterial pressure/ or diastolic blood pressure/ or blood pressure/ or systolic blood pressure/) AND (time at target.mp. or time in therapeutic range.mp. or time in target range.mp. or %blood pressure at target.mp. or proportion of blood pressure readings.mp. or blood pressure within the target over time.mp. or time spent with blood pressure controlled.mp. or time spent at blood pressure target.mp) AND (cardiovascular outcome*.mp. or cardiovascular event*.mp. or cardiovascular disease* mp or cardiovascular risk.mp or clinical outcome*.mp. or adverse clinical event*.mp. or adverse outcome*.mp.)	25/9/23	99	Exclude 73
PubMed	("hypertension"[MeSH Terms] OR "blood pressure"[MeSH Terms] OR "blood pressure determination"[MeSH Terms] OR "arterial pressure"[MeSH Terms] OR "blood pressure monitoring, ambulatory"[MeSH Terms] OR "Blood Pressure Monitors"[MeSH Terms]) AND (time in therapeutic range[Title/Abstract] OR time at target[Title/Abstract] OR time in target range[Title/Abstract] OR blood pressure at target[Title/Abstract] OR proportion of blood pressure readings[Title/Abstract] OR blood pressure within the target over time[Title/Abstract] OR time spent with blood pressure controlled[Title/Abstract]) AND ("cardiovascular outcome*"[Title/Abstract] OR "clinical outcome*"[Title/Abstract] OR "adverse clinical event*"[Title/Abstract] OR "adverse outcome*"[Title/Abstract] OR "cardiovascular event*"[Title/Abstract])	26/9/23	109	Exclude 99
Scopus	(((TITLE-ABS-KEY ("hypertension")) OR (TITLE-ABS-KEY ("systolic hypertension")) OR (TITLE-ABS-KEY ("blood pressure")) OR (TITLE-ABS-KEY ("systolic blood pressure")) OR (TITLE-ABS-KEY ("diastolic blood pressure")) OR (TITLE-ABS-KEY ("arterial pressure"))) AND(((TITLE-ABS-KEY("time at target") OR TITLE-ABS-KEY("time in therapeutic range") OR TITLE-ABS-KEY("time in target range") OR TITLE-ABS-KEY("%blood pressure at target") OR TITLE-ABS-KEY("proportion of blood pressure readings") OR TITLE-ABS-KEY("blood pressure within the target over time") OR TITLE-ABS-KEY("time spent with blood pressure controlled") OR TITLE-ABS-KEY("time spent at blood pressure target"))) AND(((TITLE-ABS-KEY("cardiovascular outcome*") OR TITLE-ABS-KEY("cardiovascular event*") OR TITLE-ABS-KEY("cardiovascular disease*") OR TITLE-ABS-KEY("cardiovascular risk ") OR TITLE-ABS-KEY("clinical outcome*") OR TITLE-ABS-KEY("adverse clinical event*") OR TITLE-ABS-KEY("adverse outcome*")))	26/9/23	75	Exclude 58

Web of Science	(TS=("hypertension" OR "systolic hypertension" OR "blood pressure" OR "systolic blood pressure" OR "diastolic blood pressure" OR "arterial pressure")) AND (TS=("time at target" OR "time in therapeutic range" OR "time in target range" OR "%blood pressure at target" OR "proportion of blood pressure readings" OR "blood pressure within the target over time" OR "time spent with blood pressure controlled" OR "time spent at blood pressure target")) AND (TS=("cardiovascular outcome*" OR "cardiovascular event*" OR "cardiovascular disease*" OR "cardiovascular risk" OR "clinical outcome*" OR "adverse clinical event*" OR "adverse outcome*"))	27/9/23	38	Exclude 21
CINAHL	(AB "hypertension" OR AB "systolic hypertension" OR AB "blood pressure" OR AB "systolic blood pressure" OR AB "diastolic blood pressure" OR AB "arterial pressure") AND (AB "time at target" OR AB "time in therapeutic range" OR AB "time in target range" OR AB "%blood pressure at target" OR AB "proportion of blood pressure readings" OR AB "blood pressure within the target over time" OR AB "time spent with blood pressure controlled" OR AB "time spent at blood pressure target") AND (AB "cardiovascular outcome*" OR AB "clinical outcome*" OR AB "adverse clinical event*" OR AB "cardiovascular event*" OR AB "cardiovascular disease*" OR AB "cardiovascular risk " OR AB "adverse outcome*")	27/9/23	47	Exclude 43

Supplementary Table 2. Summary of terminology and methodological aspects for BP-TTR determination across included studies (N=17).

Variations in terminology and methodological aspects for BP-TTR determination	N (%)
**Terminology and abbreviation**	
Time in target range, TTR	9 (53)
Time in target range, TIR	1 (6)
Time in therapeutic range, TTR	5 (29)
Time at target, TITRE	1 (6)
Proportion of on-treatment visits	1 (6)
**BP measurement modality**	
Office BP	15 (88)
Ambulatory BP	2 (12)
Home BP	1 (6)
**Target BP range ***	
BP < 150/90 mmHg	1
BP < 140/90 mmHg	2
BP < 130/80 mmHg	1
SBP within 90-140 mmHg	1
SBP within 110-130 mmHg	7
SBP within 120-130 mmHg	1
SBP within 120-140 mmHg	5
SBP < 130 mmHg	2
DBP within 70-80 mmHg	1

DBP within 60-90 mmHg	1
DBP < 80 mmHg	1
**Duration for BP-TTR determination**	
Very short term (24 hours; 13 days)	2 (12)
Short term (3 to 6 months)	5 (29)
Long term (1 to 15 years)	10 (59)
**Average number of BP readings**	
1 to 5	7 (41)
6 to 10	4 (24)
More than 10	6 (35)

* Some studies had separate target BP ranges for subgroups

BP: blood pressure; DBP: diastolic blood pressure; SBP: systolic blood pressure

Supplementary Table 3. Clinical outcomes defined in included studies.

Study	MACE (Composite)	Nonfatal MI	MI	Nonfatal stroke	Ischemic stroke	Total stroke	CV-death	HF hospitalization	All-cause mortality	Others
Buckley et al. 2023[1]		✓		✓			✓	✓		
Chen KY et al. 2023[2]	✓	●		●			●	●	●	
Chen KY et al. 2022[3]									●	✓ - composite CV death or HF hospitalization<br><br>● - CV mortality, HF hospitalization or any hospitalization
Cheng et al. 2023[4]	✓	●				●	●	●	●	
Chung et al. 2018[5]	✓						✓	✓	●	✓ - any CV disease and death<br><br>● - incident stable angina; peripheral artery disease
Doumas et al. 2017[6]									✓	
Fatani et al. 2021[7]	✓	✓	✓			✓	✓	✓		✓ - treatment-related serious adverse events (hypotension, injurious falls, bradycardia, syncope, electrolyte imbalances, acute kidney injury); composite of serious adverse events

Study	MACE (Composite)	Nonfatal MI	MI	Nonfatal stroke	Ischemic stroke	Total stroke	CV-death	HF hospitalization	All-cause mortality	Others
Fu et al. 2023[8]	✓						●		✓	
Huang et al. 2022[9]	✓						✓	✓	✓	
Kakaletsis et al. 2023[10]										✓ - unfavorable functional outcomes (disability/death) in non-stroke patients
Kario et al. 2023[11]						✓		✓		✓- coronary heart disease; aortic dissection
Kim et al. 2022[12]					✓					✓ - systemic embolism
Kodani et al. 2022[13]						✓	✓		✓	✓ - thromboembolism; transient ischemic attack;
Lin et al. 2023[14]	✓									● - composite stroke, MI, or cardiac death; composite stroke, MI, or angina pectoris; composite stroke, MI, angina pectoris, or all-cause mortality
Mahfoud et al. 2022[15]	✓		✓			✓		✓	✓	✓ - renal artery intervention; hospitalization due to heart failure, atrial fibrillation, or hypertensive crisis
Mancia et al. 2016[16]	✓		●			●	●	●	●	● - all major CV events

Study	MACE (Composite)	Nonfatal MI	MI	Nonfatal stroke	Ischemic stroke	Total stroke	CV-death	HF hospitalization	All-cause mortality	Others
Sideris et al. 2022[17]	✓									

✓ - primary outcome

● - secondary outcome

CV - cardiovascular; HF – heart failure; MACE - major adverse cardiovascular events; MI - myocardial infarction;

MACE definitions of various studies:

Cheng et al. 2023 and Chen et al 2023: the composite of nonfatal-MI, nonfatal stroke, or CV death

Chen et al 2022 and Huang et al 2022: the combined endpoint of cardiovascular disease death, aborted cardiac arrest, or heart failure hospitalization

Chung et al 2018, Mahfoud et al 2022 and Lin et al. 2023: the composite of stroke, MI, or cardiac death

Fu et al 2023: the composite of all-cause mortality plus CV rehospitalization

Huang et al 2022: composite of CV death, aborted cardiac arrest, or HF hospitalization

Lin et al 2023: composite of first stroke, MI, angina pectoris or cardiac death

Mancia et al. 2016: the composite of fatal or non-fatal myocardial infarction, sudden cardiac death, death from revascularization procedures, heart failure requiring hospitalization, and emergency procedures to prevent myocardial infarction

Sideris et al. 2022: the composite of fatal and non-fatal CV events, including incident coronary artery disease (MI or coronary revascularization procedures) or > 50% coronary artery stenosis, and stroke

Supplementary Table 4. Information on analyses used for assessing relationship between BP-TTR and cardiovascular outcomes.

Study	Association/Prediction?	TTR as continuous/categorical variable?	Analysis used	Risk assessment	BP variable included in model(s)	Was BPV included?	Comments
Buckley et al. 2023[1]	Association	Categorical	Cox proportional hazards model	Hazard ratios	1) Baseline SBP,<br>2) mean achieved SBP	No	
Chen KY et al. 2023[2]	Association	Categorical and continuous	Cox proportional hazards model	Hazard ratios	Baseline SBP	No	SBP-TTR was significantly associated with outcomes regardless of whether it was included as a continuous or categorical variable
Chen KY et al. 2022[3]	Prediction (of 5-year risk of MACE)	Categorical	Discrimination performance using Harrell's C statistic; calibration performance by Hosmer Lemeshow; reclassification performance by integrated discrimination index based on 1000 bootstrap samples of C statistic		1) Baseline SBP (reference)<br>2) Baseline SBP + last office SBP<br>3) Baseline SBP + mean achieved SBP<br>4) Baseline SBP + last office SBP + mean achieved SBP	Yes (SD of all BP measurements), model 4	All models were predictive of MACE
Cheng et al. 2023[4]	Association	Categorical	Cox proportional hazards model	Hazard ratios	SBP (all recorded values?)	No	It was not clear if all SBP values were included in the model, as SBP was measured at baseline and at every subsequent visit
Chung et al. 2018[5]	Association	Categorical and continuous	Cox proportional hazards model	Hazard ratios	1) Baseline BP<br>2) Baseline BP + mean BP	Yes (SD of all BP measurements)	BP-TTR was significantly associated with outcomes regardless of whether it was included as a continuous or categorical variable<br><br>Separate model with baseline BP + BPV was also fitted, where BP-TTR remained significantly associated with outcomes

Study	Association/Prediction?	TTR as continuous/categorical variable?	Analysis used	Risk assessment	BP variable included in model(s)	Was BPV included?	Comments
Doumas et al. 2017[6]	Association	Categorical	Generalized mixed effects model	Odds ratio	Snapshot BP control status (defined as a single on-target BP measurement during first year of follow up)	Yes for sensitivity analysis (SD of follow-up BP measurements)	
Fatani et al. 2021[7]	Association	Categorical	Multivariate logistic regression<br>Cox proportional hazards model	Odds ratio and hazards ratio	No BP variable included in model.	No	Analyses performed separately in groups of different BP categories (normotension, intermediate hypertension and established hypertension)
Fu et al. 2023[8]	Association	Continuous	Cox proportional hazards model	Hazard ratios	1) Baseline SBP<br>2) Baseline SBP + mean SBP	Yes (SD of all BP measurements), separate from mean BP	BP-TTR remained significantly associated with CV outcomes in the presence of mean SBP or BPV
Huang et al. 2022[9]	Association	Categorical and continuous	Cox proportional hazards model	Hazard ratios	1) Baseline SBP<br>2) Baseline SBP + mean SBP	Yes for sensitivity analysis (SD of all SBP measurements), separate from mean BP	BP-TTR was significantly associated with outcomes regardless of whether it was included as a continuous or categorical variable
Kakaletsis et al. 2023[10]	Association	Categorical and continuous	Cox proportional hazards model	Hazard ratios	Mean SBP	No	BP-TTR was significantly associated with outcomes regardless of whether it was included as a continuous or categorical variable
Kario et al. 2023[11]	Association	Continuous	Logistic regression	Odds ratio	1) Baseline BP<br>2) Baseline BP + mean BP	Yes (SD of all BP measurements), separate from mean BP	
Kim et al. 2022[12]	Association	Categorical	Cox proportional hazards model	Hazard ratios	Baseline office SBP	No	
Kodani et al. 2022[13]	Association	Categorical	Cox proportional hazards model	Hazard ratios	No BP variable included in model.	No	

Study	Association/Prediction?	TTR as continuous/categorical variable?	Analysis used	Risk assessment	BP variable included in model(s)	Was BPV included?	Comments
Lin et al. 2023[14]	Association	Categorical and continuous	Cox proportional hazards model	Hazard ratios	BP closest to time of event or end of follow-up	No	
Mahfoud et al. 2022[15]	Association	Categorical and continuous	Cox proportional hazards model	Hazard ratios	1) Baseline BP2) Baseline BP + last measured BP3) Baseline BP + mean BP	No	
Mancia et al. 2016[16]	Association	Continuous	Cox proportional hazards model	Hazard ratios	Baseline SBP	No	
Sideris et al. 2022[17]	Association	Categorical	Cox proportional hazards model	Hazard ratios	Baseline BP	No	
	Association	Categorical	Cox proportional hazards model	Hazard ratios	Baseline BP	Yes (coefficient of variation of office SBP across visits)	BPV had a 6% lower outcome association compared to BP-TTR for each standardized change in BP-TTR or BPV.

BP: blood pressure; BPV: blood pressure variability; DBP: diastolic blood pressure; SBP: systolic blood pressure; SD: standard deviation; TTR: time in therapeutic range

Supplementary Table 5. Different levels of BP-TTR at which reduction/increase in risk of cardiovascular events was observed.

Study	BP-TTR level at which significant risk reduction/increase was observed
Buckley et al. 2023	Significant reduction in MACE not observed until SBP-TTR >70% (HR 0.69[0.52,0.91]).
Chen et al. 2023	Biggest improvement from Q1 (0% to 22.9%) to Q2 (22.9 - 43.4%) HR 0.7[0.59,0.94], continued improvement observed in Q4 (61.9-100%).
Chen et al. 2022	Biggest change in risk reduction from Q1 (0% to <2%) to Q2 (2 to <19%) (HR 0.75[0.65,0.86]), with smaller improvement beyond (HR for SBP-TTR 38-100%: 0.71[0.60,0.82]).
Cheng et al. 2023	No significant reduction in risk until TTR >= 75% (HR 0.65[0.47,0.87]).
Chung et al. 2018	Greatest improvement from BP-TTR at 0% to < 3 months (OR 0.47[0.4,0.56]), continued improvement for BP-TTR at 3-5.9 months (i.e. BP-TTR 25% to 50%; 0.25[0.21,0.31]), then little to no improvement beyond that
Doumas et al. 2017	Compared to BP-TTR of 76% to 100%, greatest increased risk of mortality was at BP-TTR < 25%, with small differences between 50-75% and 75-100%, hence TTR>50% was considered optimal level for risk reduction.
Fantani et al. 2021	No BP-TTR categorical analysis or sensitivity/specificity analysis to determine optimum threshold.
Fu et al. 2023	Compared to BP-TTR 78-100%, there was a stepwise increased risk of all-cause mortality as BP-TTR decreased, with greatest risk at BP-TTR 0-33% (HR 1.53[1.14,2.04]). The greatest change in risk was from BP-TTR 78-100% to BP-TTR of 55-78% (HR 1.32[0.98,1.78]), with smaller increase in risk as TTR decreased.
Huang et al. 2022	Lowest risk of adverse outcomes observed in the highest TTR group (>75-100%), with no significant reduction in risk with BP-TTR < 75%.
Kakaletsis et al. 2023	No BP-TTR categorical analysis or sensitivity/specificity analysis to determine optimum threshold.
Kario et al. 2023	No significant increase in overall risk until BP-TTR < 15% (HR 0.65[0.47,0.87]), but significant increase in risk for stroke was observed from BP-TTR < 67%.
Kim et al. 2022	No further increased risk of ischemic stroke or systemic embolism observed from SBP-TTR < 69% and DBP-TTR < 77%. Receiver operator characteristic analysis showed SBP-TTR of 77% was best cutoff value for risk prediction.
Kodani et al. 2022	No significant increase in risk of CV or all-cause mortality until SBP-TTR < 50%.
Lin et al. 2023	Greatest improvement in risk reduction from BP-TTR 0-25% to BP-TTR 25-50% (HR 0.69[0.53,0.89], with smaller improvements after BP-TTR > 50%.
Mahfoud et al. 2022	Compared to a BP-TTR of > 50%, increase in risk of MACE was observed from BP-TTR 0-50% (HR 2.17[1.18,4.00]) and further increased with BP-TTR at 0% (HR 3.67 [2.12,6.38]).
Mancia et al. 2016	No further improvement in risk reduction of cardiac events, CV events and CV mortality beyond BP-TTR of 50-74%.
Sideris et al. 2022	No significant increase in risk until BP-TTR <=40% (HR 2.77[1.40,5.49]), compared to BP-TTR > 67%.

BP: blood pressure; CV: cardiovascular; DBP: diastolic blood pressure; HR: hazard ratio; MACE: major adverse cardiovascular events; SBP: systolic blood pressure; TTR: time in target range;

1. Buckley LF, Baker WL, Van Tassell BW, Cohen JB, Alkhezi O, Bress AP, Dixon DL. Systolic Blood Pressure Time in Target Range and Major Adverse Kidney and Cardiovascular Events. *Hypertension (Dallas, Tex : 1979)*. 2023;80:305-313. doi: 10.1161/HYPERTENSIONAHA.122.20141
2. Chen K, Wu Z, Shi R, Wang Q, Yuan X, Wu G, Shi G, Li C, Chen T. Longer time in blood pressure target range improves cardiovascular outcomes among patients with Type 2 diabetes: A secondary analysis of a randomized clinical trial. *Diabetes Research and Clinical Practice*. 2023;198. doi: 10.1016/j.diabres.2023.110600
3. Chen K, Li C, Cornelius V, Yu D, Wang Q, Shi R, Wu Z, Su H, Yan J, Chen T, et al. Prognostic Value of Time in Blood Pressure Target Range Among Patients With Heart Failure. *JACC: Heart Failure*. 2022;10:369-379. doi: 10.1016/j.jchf.2022.01.010
4. Cheng Y, Wang D, Yang Y, Miao Y, Shen WL, Tian J, Sheng CS. Diastolic and systolic blood pressure time in target range as a cardiovascular risk marker in patients with type 2 diabetes: A post hoc analysis of ACCORD BP trial. *Diabetes Research and Clinical Practice*. 2023;203. doi: 10.1016/j.diabres.2023.110831
5. Chung SC, Pujades-Rodriguez M, Duyx B, Denaxas SC, Pasea L, Hingorani A, Timmis A, Williams B, Hemingway H. Time spent at blood pressure target and the risk of death and cardiovascular diseases. *PLoS ONE*. 2018;13(9) (no pagination).
6. Doumas M, Tsioufis C, Fletcher R, Amdur R, Faselis C, Papademetriou V. Time in Therapeutic Range, as a Determinant of All-Cause Mortality in Patients With Hypertension. *Journal of the American Heart Association*. 2017;6. doi: 10.1161/jaha.117.007131
7. Fatani N, Dixon DL, Van Tassell BW, Fanikos J, Buckley LF. Systolic Blood Pressure Time in Target Range and Cardiovascular Outcomes in Patients With Hypertension. *Journal of the American College of Cardiology*. 2021;77:1290-1299. doi: 10.1016/j.jacc.2021.01.014
8. Fu G, Zhou Z, Jian B, Huang S, Feng Z, Liang M, Liu Q, Huang Y, Liu K, Chen G, et al. Systolic blood pressure time in target range and long-term outcomes in patients with ischemic cardiomyopathy. *American Heart Journal*. 2023;258:177-185. doi: https://dx.doi.org/10.1016/j.ahj.2022.12.011
9. Huang R, Lin Y, Liu M, Xiong Z, Zhang S, Zhong X, Ye X, Huang Y, Zhuang X, Liao X. Time in Target Range for Systolic Blood Pressure and Cardiovascular Outcomes in Patients With Heart Failure With Preserved Ejection Fraction. *Journal of the American Heart Association*. 2022;11. doi: 10.1161/JAHA.121.022765
10. Kakaletsis N, Ntaios G, Milionis H, Protogerou AD, Karagiannaki A, Chouvarda I, Dourliou V, Ladakis I, Kaiafa G, Daios S, et al. Time of blood pressure in target range in acute ischemic stroke. *Journal of Hypertension*. 2023;41:303-309. doi: 10.1097/HJH.0000000000003331
11. Kario K, Tomitani N, Okawara Y, Kanegae H, Hoshide S. Home systolic blood pressure time in therapeutic range and cardiovascular risk: the practitioner-based nationwide J-HOP study extended. *Hypertension Research*. 2023.
12. Kim M, Cho MS, Nam GB, Do U, Kim J, Choi KJ. Controlled Level and Variability of Systolic Blood Pressure on the Risk of Thromboembolic Events in Atrial Fibrillation and Hypertension. *The American journal of cardiology*. 2022;180:37-43. doi: 10.1016/j.amjcard.2022.06.036
13. Kodani E, Inoue H, Atarashi H, Okumura K, Suzuki S, Yamashita T, Origasa H. Impact of Systolic Blood Pressure Time in Target Range on Adverse Events in Patients With Nonvalvular Atrial Fibrillation (from the J-RHYTHM Registry). *American Journal of Cardiology*. 2022;180:52-58. doi: https://dx.doi.org/10.1016/j.amjcard.2022.06.045
14. Lin Z, Xiao Z, Chen W, Xu W, Huang C, Xie J, Jin M, Wei X, He S, Xie Z, et al. Association of long-term time in target range for systolic blood pressure with cardiovascular risk in the elderly: A Chinese veteran cohort study. *European Journal of Preventive Cardiology*. 2023;30:969-977. doi: 10.1093/eurjpc/zwad083

15. Mahfoud F, Mancia G, Schmieder RE, Ruilope L, Narkiewicz K, Schlaich M, Williams B, Ribichini F, Weil J, Hsien-Li K, et al. Cardiovascular Risk Reduction After Renal Denervation According to Time in Therapeutic Systolic Blood Pressure Range. *Journal of the American College of Cardiology*. 2022;80:1871-1880. doi: 10.1016/j.jacc.2022.08.802
16. Mancia G, Kjeldsen SE, Zappe DH, Holzhauer B, Hua TA, Zanchetti A, Julius S, Weber MA. Cardiovascular outcomes at different on-treatment blood pressures in the hypertensive patients of the VALUE trial. *European heart journal*. 2016;37:955-964. doi: 10.1093/eurheartj/ehv633
17. Sideris K, Andrikou I, Thomopoulos C, Tatakis F, Kariori M, Manta E, Kalos T, Soulaidopoulos S, Drogkaris S, Konstantinidis D, et al. Blood pressure control measures and cardiovascular outcomes: a prospective hypertensive cohort. *Blood Pressure*. 2022;31:228-235. doi: 10.1080/08037051.2022.2118662
